# Time Spent Sitting Is Associated with Changes in Biomarkers of Frailty in Hospitalized Older Adults: A Cross Sectional Study

**DOI:** 10.3389/fphys.2017.00505

**Published:** 2017-07-31

**Authors:** Jair S. Virtuoso Júnior, Liliane B. Roza, Sheilla Tribess, Joilson Meneguci, Edmar L. Mendes, Maycon S. Pegorari, Flávia A. Dias, Darlene M. dos Santos Tavares, Jeffer E. Sasaki

**Affiliations:** ^1^Postgraduate Program in Healthcare, Federal University of Triângulo Mineiro Uberaba, Brazil; ^2^Department of Sport Sciences, Postgraduate Program in Physical Education, Federal University of Triângulo Mineiro Uberaba, Brazil; ^3^Physiotherapy Course, Federal University of Amapá Macapá, Brazil; ^4^Department of Nursing in Community Health and Education, Postgraduate Program in Healthcare, Federal University of Triângulo Mineiro Uberaba, Brazil

**Keywords:** sedentary behavior, frailty, ROC curve, inflammation, older adults

## Abstract

**Background:** Sedentary behavior has gained prominence in the literature as a risk factor for health and mortality independent of physical activity level; however, little is known about the relationship of sedentary behavior with frailty in older adults. The aim of this study was to investigate if time spent sitting can be used as a discriminator of frailty in older hospitalized persons.

**Methods:** The study included 162 hospitalized inpatients aged ≥60 years. Blood samples were taken for analyzing leukocyte counts and serum concentrations of C-reactive protein (CRP). Participants also answered a questionnaire about time spent sitting. Frailty was determined from a combination of CRP concentration and leukocyte count. Receiver operating characteristic (ROC) curves were constructed to analyse the predictive power and cut-points for time spent sitting and the presence of frailty.

**Results:** The areas under the ROC curves indicated that time spent sitting was an independent indicator of frailty (area under curve >0.6). The cut-off points for time spent sitting as an indicator of frailty were >257 min/day for men and >330 min/day for women.

**Conclusions:** Time spent sitting is associated with biomarkers of frailty in persons aged ≥60 years, indicating a need for interventions aimed at reducing sedentary behavior in this age group.

## Introduction

It has been estimated that the number of older adults worldwide will reach 1 billion in the next 10 years (United Nations Population Fund, 2016). Concomitant with this increase, there will be an expansion in the use of technology, including appliances, automobiles, smartphones, and Internet. This will contribute to higher prevalence of time spent in sedentary behavior across the different age groups (Owen et al., 2010). Nevertheless, older adults will likely continue to be the segment of the population with the highest rates of sedentary behavior (Matthews et al., 2008), as they usually present with comorbidities that limit their activities of daily life.

Long periods spent in sedentary behavior are reportedly associated with elevated inflammatory state (Healy et al., 2011; Allison et al., 2012; Gennuso et al., 2013; Hamer et al., 2013; León-Latre et al., 2014; Parsons et al., 2017). Inflammation contributes to disorders such as cardiovascular disease (Emerging Risk Factors Collaboration et al., 2010), diabetes (Marques-Vidal et al., 2012), cognitive decline (Metti et al., 2014), and frailty (Gale et al., 2013).

Frailty is an important geriatric syndrome characterized by substantial declines in the function of multiple organ systems, functional capacity and higher risk for mortality (Fried et al., 2009; Li et al., 2011; Abizanda et al., 2013). Because frailty is associated with adverse health outcomes, its early identification could facilitate interventions aimed at minimizing such problems (Tribess et al., 2012). One way to diagnose frailty is examining the combination of inflammatory biomarkers, such as the serum concentration of C-reactive protein (CRP) and leukocyte count (Li et al., 2011). Elevated inflammatory state contributes to the decrease of muscle mass, strength, power, and motor performance, aspects that play an important role in the pathogenesis of frailty (Chen et al., 2014).

Due to overlapping of primary and secondary effects of aging, older adults represent the age group most vulnerable to adverse health effects. This place older adults as a susceptible group to hospitalization, wherein diagnosis of the frailty is often difficulty because of associated clinical conditions. Although, it has been shown that sedentary behavior is associated with high values of inflammatory biomarkers, limited evidence is available on the association of time spent in sedentary behavior and such biomarkers. This study aimed to analyse time spent sitting as an indicator of the presence of frailty, indicated by increased levels of inflammatory biomarkers, in hospitalized older adults.

## Materials and methods

### Study sample

This cross-sectional study is part of the research project titled “Prevalence and associated factors of frailty in older adults of a university hospital.”

The study sample comprised 1,455 persons aged 60 years or more of both sexes who were inpatients of medical and surgical wards of a university hospital from April 2013 to March 2014. The required sample size was calculated by estimating the prevalence of frailty in older adults, which was identified as 30% (Khandelwal et al., 2012). After calculation of the 95% confidence interval and a tolerable error of 5%, the required sample was estimated to be 168 study subjects.

The inclusion criteria were as follows: (1) age over 60 years; (2) agreement to participate in the study by signing an informed consent form; (3) achieving the minimum score in the Mini-Mental State Examination (Folstein et al., 1975) according to level of education specified by the criteria of Bertolucci et al. (1994); and (4) ability to walk.

Exclusion criteria were as follows: absence of serious sequelae of stroke such as localized loss of strength, aphasia, or other speech disorders that would prevent study assessments; presence of Parkinson's disease with severe impairment of motor function, speech, ability to communicate, or emotional issues that would prevent study assessments; severe deficits of vision and/or hearing that would substantially hinder communication; participation in the study during previous hospitalization; and being in the terminal stage of an illness.

### Instruments and procedures for data collection

Participants were asked to respond to a structured questionnaire administered in the form of a face-to-face interview. The questionnaire assessed relevant economic and social factors, functional disability, and sedentary behavior. Subsequently, participants provided a blood sample for determination of inflammatory biomarkers of frailty, namely C-reactive protein (CRP) and leukocyte count. Data collection was carried out by 12 appropriately trained researchers in the health field.

#### Economic and social variables

A self-report instrument developed by our Research Group on Public Health was used to gather data on sex (male/female), age (60–69 years, 70−79 years, and 80 years or more) marital status (single, married or living with a partner, and widowed, separated, or divorced), living arrangements (living alone and with others), and monthly personal income (income less or greater than one minimum wage).

#### Functional disability

Functional disability was assessed by self-reported ability to carry out basic activities of daily living (BADL) and instrumental activities of daily living (IADL). The Brazilian versions of the “Index of Independence in Activities of Daily Living” (Lino et al., 2008) and “Scale of Instrumental Activities of Daily Living” (Santos and Virtuoso-Júnior, 2008) were used, respectively.

#### Sedentary behavior

Time spent sitting was assessed with the following questions: “How long, in total, did you spend sitting during a weekday prior to hospitalization?” and “How long, in total, did you spend sitting on a weekend day prior to hospitalization?” These questions are similar to those that evaluate time spent sitting in the International Physical Activity Questionnaire (Rosenberg et al., 2008), according to another study conducted in Brazil (Santos et al., 2017). The overall sitting time in minutes/day was determined by calculating the weighted mean of the time spent sitting on a weekday and on a weekend day with the following formula:

(1) $$\begin{array}{l} \text{Overall time spent sitting}=(({\text{time spent sitting on a weekday}}^{*}5)\\ \;+\;({\text{time spent sitting on a weekend day}}^{*}2))/7. \end{array}$$

#### Frailty

Frailty was assessed by serum concentrations of the inflammatory biomarkers C-reactive protein (CRP) and leukocyte count. Two blood samples were obtained: the first tube without anticoagulant, for the determination of serum CRP concentration, and the second with ethylenediamine tetraacetic acid for the overall leukocyte count.

Serum CRP concentrations were measured by an immunoturbidimetry method using a Cobas Integra 400-Plus (Roche Diagnostics, Basel, Switzerland), and the overall white blood cell count with XE2100-D equipment (Roche Diagnostics). The cut-off values for frailty were CRP>2.6 mg/dL (Puzianowska-Kuźnicka et al., 2016) and white cell count >9290 mm^3^ (Bovill et al., 1996), which corresponded to the 4th quartile. If both values were abnormal according to these criteria, the participant was classified as frail, whereas if one or both were normal they were classified as non-frail.

### Data analysis

Data were double entered on a Microsoft Office 2007 Excel spreadsheet. Statistical analyses were performed with the Statistical Package for Social Sciences software (SPSS), version 20.0, and Medcalc, version 11.4.4. The c^2^-test was used to compare social and economic variables and functional disability according to the presence of frailty. Cut-points for time spent sitting and their predictive power for presence of frailty were identified by receiver operating characteristic curves (ROC) as well as their sensitivity and specificity values. The larger the area under the ROC curve, the greater the power of cut-points for identifying presence of frailty. The lower limit of the area under of the ROC for accepting the cut-points as predictive of frailty was set at 0.60 (Schisterman et al., 2001) with a confidence interval (CI) of 95%. Cut-points for time spent sitting as a predictor of frailty were determined after calculating sensitivity and specificity values. The significance level was set at 5% (*p* ≤ 0.05).

### Ethical considerations

This study was in accordance with the Helsinki declaration and ethical principles of the Resolution No. 466 of December 12, 2012 of the National Health Council from Brazil. The study protocol was approved by the Ethics Committee in Research with Human Beings of the Federal University of Triângulo Mineiro (Protocol number No. 2511/2012).

## Results

Of the 168 participants, 57.1% (*n* = 96) were male and 64.3% (n = 108) between 60 and 69 years old. Most participants were married or living with partners (61.3%; *n* = 103), not living alone (84.5%; *n* = 142), and had monthly incomes of less than one minimum wage (65.5%; *n* = 110). Regarding their functional capacity, 6.0% (*n* = 10) were scored as dependent for BADL, whereas 65.5% (*n* = 110) were scored as dependent for IADL (Table 1).

**Table 1:** Distribution of social and economic variables, functional disability, and sedentary behavior in the sample of older inpatients.

**Variable**	**Overall**		**Not frail**		**Frail**		p^*^
	**%**	***n***	**%**	***n***	**%**	***n***	
**GENDER**							
Male	57.1	96	52.8	75	80.8	21	0.008
Female	42.9	72	47.2	67	19.2	5	
**AGE GROUP**							
60–69 years	64.3	108	66.9	95	50.0	13	0.147
70–79 years	30.4	51	28.9	41	38.5	10	
80 years or more	5.4	9	4.2	6	11.5	3	
**MARITAL STATUS**							
Single	3.0	5	3.5	5	0.0	0	0.487
Married or living with spouse or partner	61.3	103	59.9	85	69.2	18	
Widowed or separated, divorced	35.7	60	36.6	52	30.8	8	
**LIVING ARRANGEMENT**							
Alone	15.5	26	14.8	21	19.2	5	0.565
Accompanied	84.5	142	85.2	121	80.8	21	
**INCOME**							
>1 min wage	34.5	58	33.1	47	42.3	11	0.364
<1 min wage	65.5	110	66.9	95	57.7	15	
**BASIC ACTIVITIES OF DAILY LIVING**							
Independent	94.0	158	95.8	136	84.6	22	0.027
Dependent	6.0	10	4.2	6	15.4	4	
**INSTRUMENTAL ACTIVITIES OF DAILY LIVING**							
Independent	34.5	58	36.6	52	23.1	6	0.182
Dependent	65.5	110	63.4	90	76.9	20	
**SEDENTARY BEHAVIOR**							
<240 min/day	58.3	98	62.0	88	38.5	10	0.025
≥240 min/day	41.7	70	38.0	54	61.5	16	

* *χ2-test. One minimum salary/month = $260.00*.

The prevalence of frailty was 15.5%, being more frequent in men and in those who were dependent for BADL (Table 1). The median time spent sitting was 231 min/day for men and 223 min/day for women. The time spent sitting was confirmed as a discriminator of frailty, with areas under the ROC > 0.60.

**Figure 1:**
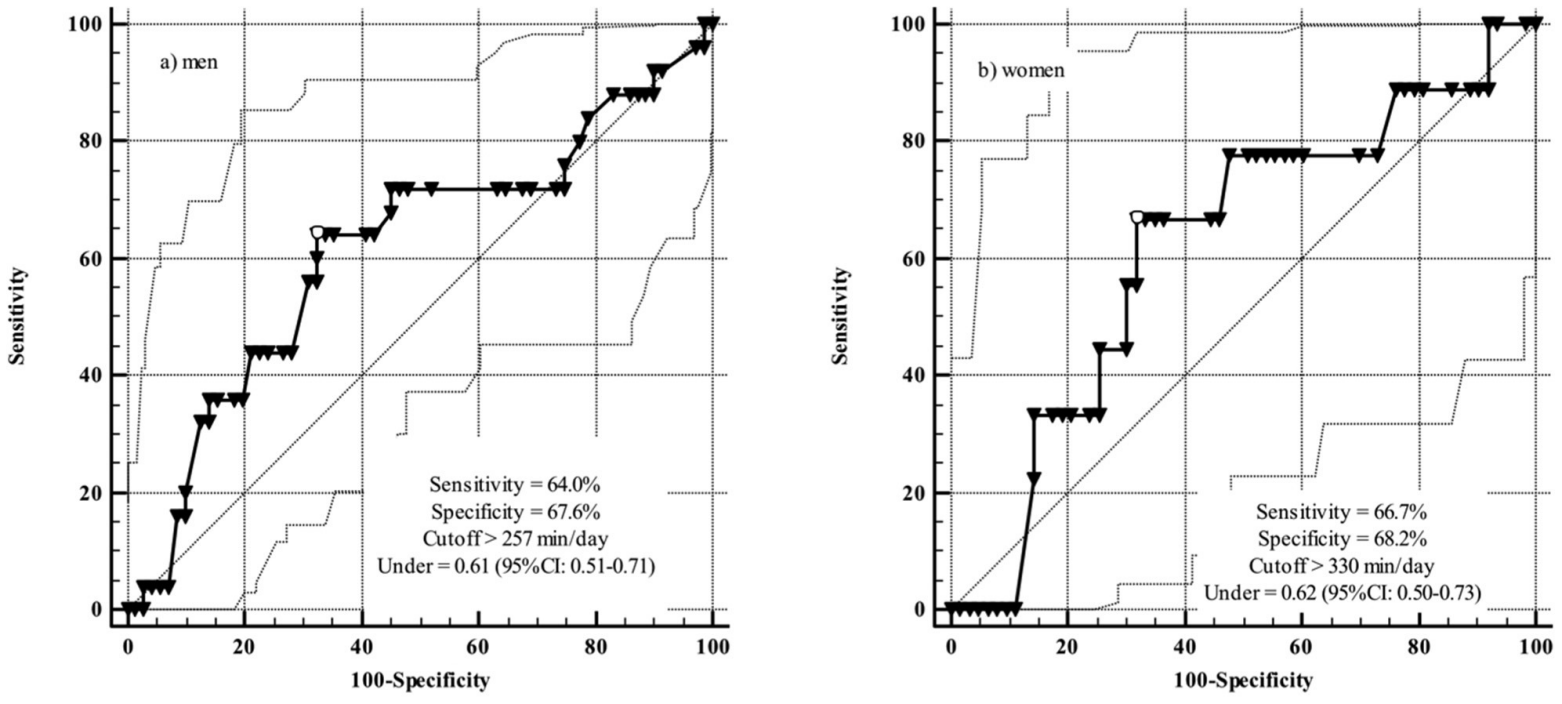
Sitting time cut-points for discriminating frailty in older adults.

The areas under the ROC curve were 0.61 for men (CI: 0.51−0.71) and 0.62 for women (CI: 0.50−0.73). Figure 1 shows the sensitivity and specificity values associated with the aforementioned areas under the ROC. The cut-points for time spent sitting as a predictor of frailty were >257 min/day and >330 min/day for men and women, respectively.

## Discussion

This study aimed to identify the predictive power of time spent sitting as a discriminator of frailty in hospitalized older adults. Previous studies have highlighted that prolonged sitting time is associated with greater vulnerability to adverse health outcomes in older persons; these include metabolic syndrome (Gardiner et al., 2011), reduced muscle strength (Hamer and Stamatakis, 2013), excessive body weight (Gómez-Cabello et al., 2012), and increased risk of mortality from all causes (Pavey et al., 2015; Lee, 2016). However, little is known concerning the association between sedentary behavior and frailty (Blodgett et al., 2015; da Silva Coqueiro et al., 2016).

In this study, a greater proportion of men were considered frail in comparison to women. One possible explanation would be a greater engagement in light intensity physical activities by older women compared to men. Sedentary behavior is usually replaced with light intensity physical activity (Buman et al., 2010). In this regard, women tend to perform more domestic activities than men, thus presenting less exposure to sedentary behavior (Murphy et al., 2013).

This study determined frailty status based on simultaneous abnormalities in two inflammatory biomarkers, namely serum concentrations of CRP and leukocyte count (Li et al., 2011). These biomarkers have been associated with frailty and also with morbidity and mortality in older adults (Willems et al., 2010; Kim et al., 2013; Salazar et al., 2014). The association between time spent in sedentary behavior and increased CRP concentrations and leukocyte counts identified in large population studies (Healy et al., 2011; Pinto Pereira et al., 2012; León-Latre et al., 2014) has received considerable attention in recent years. A study with Americans found that sedentary behavior was associated with limited mobility and that participants with reduced mobility had higher CRP concentrations and leukocyte counts than those with unrestricted mobility (Loprinzi, 2013). A linear association of time spent sitting at work with CRP concentration and leukocyte count has been reported in a study with Spanish workers (León-Latre et al., 2014). It has also been shown that in older persons from UK, longer television viewing times were associated with higher CRP concentrations; this association remains significant even after controlling for confounding variables (Hamer et al., 2013). Similar findings have been reported in older American individuals (Gennuso et al., 2013).

The relationship between sedentary behavior and dysfunction of the immune system might be explained by the reduced muscle contraction, which can result in increased muscle glucose and decreased insulin sensitivity (Charansonney and Després, 2010; Charansonney, 2011). Sparing glucose is then metabolized by the liver into fat and stored in central adipocytes (Meneguci et al., 2015). Adipose tissue, in turn, releases a variety of synthesized proteins termed adipokines (Charansonney, 2011), among which, resistin is positively correlated with the immune and inflammatory system (Kunnari et al., 2006). Increases in resistin concentrations induce increases in leukocyte counts and concentrations of C-reactive protein (Kunnari et al., 2006).

Although, participants from this study were medical or surgical clinic inpatients of a university hospital, they were all able to walk independently. It is also important to emphasize that participants reported time spent sitting from the period prior to hospitalization, not during hospitalization. Hospitalization can increase the risk of deleterious health effects in older adults (Graf, 2006). Thus, appropriate intervention strategies for interrupting sedentary behavior are necessary in the hospitalization period. Interruption of prolonged periods of sedentary behavior can contribute to reduction of adverse health outcomes (Bailey and Locke, 2015; Júdice et al., 2015). A recent randomized study showed that 2 min of moderate walking for every 20 min spent sitting is associated with a reduction in postprandial blood glucose concentrations (Bailey and Locke, 2015). Interruptions in sedentary behavior have been associated with positive metabolic effects, such as smaller waist circumference and body mass index, as well as lower serum triglyceride and glucose concentrations (Healy et al., 2008). Breaks in sedentary behavior also protect against frailty and are associated with positive changes in concentrations of C-reactive protein (Healy et al., 2011). Additionally, a recent study showed that breaks in sedentary behavior are positively associated with components of physical fitness (Sardinha et al., 2015); and the latter has been directly related to frailty (Fried et al., 2001). In view of this, the cut-points presented in this study may be used to identify those individuals who need special attention for avoiding the frailty syndrome. In addition, the cut-points can be used in interventions as target values for guiding reductions in sedentary behavior in older adults.

The present study has limitations. Diagnosis of frailty was based on only two inflammatory biomarkers, which in hospitalized older adults may be altered for many different reasons, including acute infections. Thus, it is likely that some participants may have been misclassified by the criteria herein adopted for determining frailty status. Another limitation was the lack of an objective method to assess sedentary behavior. The use of an accelerometer would have resulted in more accurate measures of sedentary behavior, as older adults might present difficulties recalling their daily routine. The sampling procedure may also have introduced bias in selecting participants. Ideally, the stratification of participants by reasons for hospital admission as well as type of medications used would have minimized selection bias in this study. Finally, the cross-sectional design of the study is a limitation that precludes conclusions about cause and effect.

In conclusion, this study demonstrated that time spent sitting >257 min/day for men and >330 min/day for women are discriminators for the presence of frailty in older persons. While our results need to be interpreted with caution, they do support that sedentary behavior may be related to frailty in older adults, defined as elevated inflammatory biomarkers in this study. Future studies are needed to examine the relationship between sedentary behavior and frailty in hospitalized older adults. These studies should follow-up participants after hospital discharge in order to identify their susceptibility to adverse events and, at the same time, examine predictive validity of inflammatory biomarkers for frailty.

## Author contributions

JV and LR initiated the article and wrote the first draft. ST, JM, EM, MP, FD, DD, and JS helped writing and provided input into subsequent revisions.

### Conflict of interest statement

The authors declare that the research was conducted in the absence of any commercial or financial relationships that could be construed as a potential conflict of interest.
